# *De novo* synthesis of L-2-aminobutyric acid in *Escherichia coli* based on multi-layered metabolic engineering strategies

**DOI:** 10.1016/j.synbio.2026.01.004

**Published:** 2026-01-15

**Authors:** Zhenqiang Zhao, Yizheng Liu, Rongshuai Zhu, Fengyu Yang, Zhifei Liu, Jiajia You, Xuewei Pan, Jianming Yang, Zhiming Rao

**Affiliations:** a College of Life Sciences, Qingdao Agricultural University, Qingdao, China; b The Key Laboratory of Industrial Biotechnology, Ministry of Education, School of Biotechnology, Jiangnan University, Wuxi, 214122, China; c Yixing Institute of Food and Biotechnology Co., Ltd, Yixing, Jiangsu, 214200, China

**Keywords:** L-2-aminobutyric acid, Quorum sensing, Metabolic modeling, redox cofactor balance, Metabolic engineering

## Abstract

L-2-Aminobutyric acid (L-2-ABA) is a non-proteinogenic amino acid and an important chiral intermediate widely used in pharmaceuticals and fine chemicals. However, its fermentative production is limited by intermediate toxicity and imbalanced metabolic flux. In this study, *Escherichia coli* was systematically engineered for efficient *de novo* synthesis of L-2-ABA using a multi-layer metabolic engineering strategy. A quorum-sensing–based dynamic control circuit was introduced to decouple cell growth from 2-oxobutyric acid formation, thereby alleviating precursor toxicity and improving flux coordination. Combined with optimization of the L-2-ABA conversion pathway, model-guided carbon flux redistribution, cofactor regeneration, and tuning of global transcriptional regulation, a high-performance production strain was obtained without the need for antibiotics or inducers. The final engineered strain ABA40 achieved 45.3 g/L L-2-ABA with a yield of 0.31 g/g glucose in a 72 h fed-batch fermentation. This study demonstrates the effectiveness of dynamic and integrated metabolic engineering strategies for the biosynthesis of non-natural amino acids.

## Graphical abstract

**Figure:**
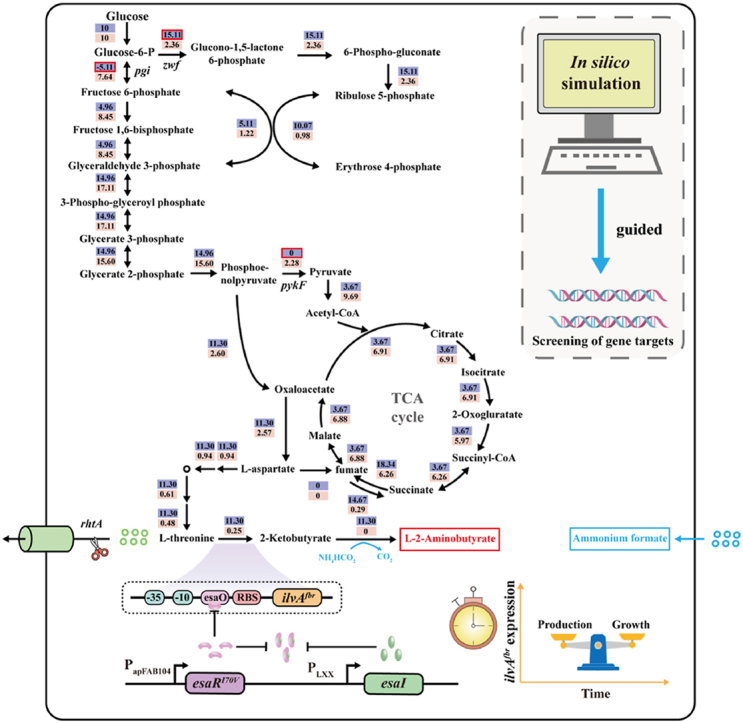

## 1 Introduction

L-2-Aminobutyric acid (MW 103.2, L-2-ABA), as a non-proteinogenic amino acid, could be used as raw chemicals and pharmaceutical intermediates [1]. It has also been reported to inhibit the transmission of neurological signals and to stimulate cellular metabolism in the brain. In addition, (S)-2-aminobutyramide, generated by the amidation reaction of L-2-ABA, is widely used in the manufacture of antiepileptic medicines and for treating tuberculous meningitis [2,3]. With the increasing demand for related pharmaceuticals, the commercial value of 2-aminobutyric acid has attracted growing attention.

The production of L-2-ABA could be categorized into chemical and biological methods [4,5]. The chemical synthesis of L-2-ABA involves harsh catalytic conditions and low yield, leading to its gradual replacement by biological methods. The typical microbial biosynthetic pathway involves the conversion of l-threonine to 2-oxobutyric acid (2-OBA) catalyzed by threonine deaminase, followed by the reduction of 2-OBA to L-2-ABA through amino acid transaminases or dehydrogenases [[6], [7], [8]]. Therein, the reversibility of the transamination reaction limits the increase in the yield of L-2-ABA and the incorporation of amino donors leads to the production of by-products. Dehydrogenases (e.g., leucine dehydrogenase, phenylalanine dehydrogenase, alanine dehydrogenase) can directly incorporate ammonium ions (NH_4_^+^) into 2-OBA, thereby avoiding the consumption of amino donors and the formation of by-products [[9], [10], [11]]. In addition, balancing cell growth with 2-OBA production might become the key to improve L-2-ABA production, due to the toxic effect of 2-OBA on cells [12].

Tunable quorum-sensing (QS) circuits that sense cell density have been widely used to dynamically coordinate growth and production [13,14]. For example, in the Esa quorum-sensing regulatory system, N-acyl-homoserine lactone (AHL) molecules gradually accumulate with increasing biomass and bind to the transcriptional repressor EsaR, preventing its association with the target DNA sequence and thereby relieving repression of downstream gene expression [15]. This strategy effectively avoids the problem of cell growth restriction due to premature synthesis of toxic intermediates. Furthermore, maintaining an adequate supply of the precursor l-threonine is essential for the *de novo* biosynthesis of L-2-ABA. Previously, a high l-threonine-producing strain, THR47, was constructed through a multidimensional engineering strategy, which ensured a stable precursor pool to support efficient 2-OBA formation [16]. With the precursor supply adequately ensured, further improvement in L-2-ABA production was primarily constrained by the intracellular redox balance, as leucine dehydrogenase (LeuDH), the key enzyme that catalyzes the conversion of 2-OBA to L-2-ABA, depends on NADH as a reducing cofactor [17]. Increasing intracellular NADH by converting NADPH to NADH or implementing NADH-recycling systems could alleviate this constraint [18]; however, such strategies must be balanced against the substantial NADPH demand of the l-threonine biosynthetic pathway to avoid compromising precursor formation.

In this study, an engineered strain for the *de novo* biosynthesis of L-2-ABA was constructed using the high l-threonine-producing strain THR47 as the chassis. To maximize precursor supply and achieve efficient carbon flux distribution, the expression of l-threonine deaminase was dynamically regulated through an Esa-QS circuit, which enabled coordinated control of growth and 2-OBA formation. Furthermore, the L-2-ABA biosynthetic pathway was established and optimized by fine-tuning the activity of LeuDH from *Exiguobacterium sibiricum*. To overcome cofactor limitations, NADH availability was improved through cofactor engineering. Subsequently, key engineering targets were predicted and validated through genome-scale metabolic network modeling. The l-threonine transport system was optimized, the 2-OBA degradation pathway was dynamically regulated, and transcription factor engineering was employed to reshape carbon flux distribution and enhance cellular robustness, collectively improving L-2-ABA biosynthetic efficiency. As a result, the engineered strain *E. coli* ABA40 achieved an L-2-ABA titer of 45.3 g/L with a yield of 0.31 g/g glucose during fermentation, representing one of the highest titers reported for fermentative L-2-ABA production to date.

## 2 Materials and methods

### 2.1 Strains, plasmids, and general reagents

The chassis strain used in this study was the l-threonine-producing strain *E*. *coli* THR47. The genotypes of all engineered strains (ABA01–ABA40) are listed in supporting information Table S1. For functional verification of tyrosine aminotransferase and leucine dehydrogenase, the low-copy plasmid pTH18kr was employed for heterologous expression. *E*. *coli* DH5α was used for plasmid propagation and construction. Oligonucleotide primers and synthetic genes were provided by Sangon Biotech Co., Ltd. (Shanghai, China) and verified by sequencing. All chemical reagents were of analytical grade or higher: L-2-ABA, 2-OBA, l-threonine, ammonium formate, and glucose were purchased from Shanghai Chemical Reagent Co., Ltd., Sinopharm (Shanghai, China). All strains were stored in 25 % (v/v) glycerol at −80 °C for long-term preservation.

### 2.2 Genome engineering

Genomic integration of *leuDH*^K72A^ was performed using a CRISPR/Cas9-assisted homologous recombination strategy, with integration at the yedS locus as an example. The *yedS* locus is annotated as a pseudogene in the KEGG database and is considered a non-essential genomic locus in *E. coli*. Insertion of heterologous genes at this site is unlikely to affect host growth or central metabolism and is therefore commonly used for stable chromosomal integration in metabolic engineering studies.

Firstly, the pRedCas9 plasmid carrying the Cas9 nuclease and the λ-Red recombination system was first introduced into *E. coli* cells and cultivated at 30 °C. Expression of the λ-Red recombination machinery was induced by the addition of IPTG to a final concentration of 1 mM. A single-guide RNA (*sg*RNA) targeting the *yedS* locus was designed and cloned into the pGRB vector. Meanwhile, a donor DNA fragment containing the *leuDH*^K72A^ gene flanked by approximately 400–800 bp upstream and downstream homologous arms was constructed by fusion PCR. The donor DNA and the pGRB-*sg*RNA plasmid were co-electroporated into competent cells harboring pRedCas9. After electroporation, cells were recovered at 30 °C and 220 rpm for 2 h and subsequently plated on LB agar supplemented with spectinomycin and ampicillin. Correct integrants were verified by colony PCR. The pGRB-*sg*RNA and pRedCas9 plasmids were subsequently cured using temperature-sensitive cultivation and antibiotic counter-selection, yielding marker-free and plasmid-free engineered strains [19].

### 2.3 Genome-scale metabolic modeling and computational strain design

All computational analyses were performed using the COBRA Toolbox v3.0 in MATLAB R2022a, with Gurobi 12.0 as the optimization solver. The genome-scale model iML1515 was used as the modeling framework. To account for the non-native L-2-ABA pathway, the LeuDH-catalyzed reaction (LEUDH_2abut: 2obut_c + nh4_c + nadh_c -> 2abut_c + nad_c + h2o_c) and a sink reaction (2abut_c ->) were manually introduced into the model. For flux balance analysis (FBA), simulations were conducted under two scenarios: (i) maximal growth with unconstrained biomass flux, and (ii) growth-limited conditions with biomass flux fixed at 0.1 h^−1^. In both cases, the glucose uptake rate was constrained to 10 mmol·gDW^−1^·h^−1^, and oxygen uptake was left unconstrained.

Based on this modified model, *in silico* design was carried out using both OptKnock and OptGene. OptKnock was implemented in the COBRA Toolbox as a bilevel linear programming problem. OptGene employed a genetic algorithm to explore reaction knockout strategies via iterative selection, crossover, and mutation. The genetic algorithm was executed with a population size of 400, 400 generations, and up to five knockouts allowed per design. The same boundary conditions as in the FBA simulations were applied. The knockout candidates predicted by the algorithms were subsequently prioritized for experimental validation.

### 2.4 Measurement of intracellular NADH/NADPH ratio

Cells were harvested after 24 h of shake-flask cultivation, rapidly chilled, and collected by centrifugation at 4 °C. Intracellular reduced cofactors NADH and NADPH were extracted under alkaline conditions and neutralized prior to analysis. NADH and NADPH levels were quantified using the NAD^+^/NADH and NADP^+^/NADPH colorimetric assay kits (BioVision) according to the manufacturer's instructions. The measured values were normalized to biomass (OD_600_), and the intracellular redox state was evaluated by calculating the NADH/NADPH ratio [20].

### 2.5 Real-time PCR

Total RNA was extracted from shake-flask-cultivated strains using a commercial bacterial RNA extraction kit (RNAprep Pure Cell/Bacteria Kit, TIANGEN, Beijing, China). Reverse transcription was performed using HiScript III RT SuperMix for qPCR (+ gDNA wiper) (Vazyme, Nanjing, China) to remove residual genomic DNA and synthesize cDNA. Real-time quantitative PCR was carried out using ChamQ Universal SYBR qPCR Master Mix (Vazyme, Nanjing, China) on a real-time PCR system according to the manufacturer's instructions. The 16S rRNA gene was used as the internal reference for normalization. Relative transcription levels of chromosomally integrated *leuDH*^K72A^ were calculated using the 2^−ΔΔCt^ method. Primer sequences used for RT-qPCR are listed in Supplementary Table S2.

### 2.6 Fed-batch fermentation in a 5-L bioreactor

Fed-batch fermentation was carried out in a 5 L bioreactor (T&J Bioengineering, Shanghai, China). A single colony was first inoculated into a 50 mL flask containing 10 mL of the first seed medium and cultivated at 37 °C and 220 rpm for 12 h. Subsequently, 2 % (v/v) of the culture was transferred into a 500 mL flask containing 100 mL of the same seed medium and incubated under identical conditions for another 12 h. The resulting first seed culture was then inoculated at 5 % (v/v) into the 5 L bioreactor containing the second seed medium. When the optical density (OD_600_) of the culture reached approximately 10.0–12.0, the excess broth was discarded, leaving 400 mL of culture. This culture was supplemented with 1.6 L of the third fermentation medium to achieve an effective inoculum ratio of 20 % (v/v). This inoculum ratio was selected to shorten the lag phase and facilitate rapid establishment of the production culture. During fermentation, the pH was maintained at 7.0 ± 0.05 by automatic addition of ammonia water or 0.5 M H_2_SO_4_. Dissolved oxygen was maintained at 30 ± 2 % saturation by adjusting the agitation speed (200–800 rpm) and aeration rate (1–2 vvm). For the feeding phase, concentrated glucose (600 g/L) and ammonium formate (100 g/L) solutions were prepared separately. Feeding was initiated when the residual glucose concentration in the bioreactor dropped below 5 g/L. During the establishment of the glucose feeding strategy in preliminary fermentation batches, residual glucose concentrations were monitored every 2 h, and stage-dependent glucose consumption patterns were summarized to optimize the feeding rate. Based on this strategy, the glucose feeding rate was adjusted during fed-batch fermentation to maintain the residual concentration between 0 and 5 g/L. Ammonium formate was supplemented using a timed manual pulse-feeding strategy. Ammonium formate feeding was initiated after 10 h of fermentation, with 40 mL added every 8 h, aiming to maintain the in-broth ammonium formate concentration at approximately 3–5 g/L, while avoiding inhibitory effects from sudden increases. The fermentation process lasted for 72 h. Samples were collected every 4 h to determine OD_600_, residual glucose, organic acids, 2-OBA, and L-2-ABA concentrations.

The compositions of the media (per liter) were as follows: (i) First seed medium: 10 g peptone, 10 g NaCl, 5 g yeast extract. (ii) Second seed medium: 20 g glucose, 5 g (NH_4_)_2_SO_4_, 2 g KH_2_PO_4_, 1 g MgSO_4_·7H_2_O, 2 g yeast extract. (iii) Third fermentation medium: 20 g glucose, 5 g (NH_4_)_2_SO_4_, 2 g KH_2_PO_4_, 1 g MgSO_4_·7H_2_O, 2 g yeast extract, 5 g corn steep liquor, 10 mg thiamine, 10 mg biotin, 20 mg FeSO_4_, and 10 mg MnSO_4_.

### 2.7 Analytical methods

Cell growth was monitored by measuring the optical density of the culture at 600 nm (OD_600_) using a UV–visible spectrophotometer (Shimadzu, Japan). Glucose concentration was determined with a Sieman S-10 glucose analyzer (Sieman, China). The concentrations of 2-OBA were quantified by high-performance liquid chromatography (HPLC, Agilent 1260 Infinity, USA) equipped with an Aminex HPX-87H column (Bio-Rad, USA) and a refractive index detector. The separation was performed with 5 mM H_2_SO_4_ as the mobile phase at a flow rate of 0.6 mL/min and a column temperature of 55 °C.

The concentrations of l-threonine and L-2-ABA were determined after pre-column derivatization with *o*-phthaldialdehyde (OPA), using a reversed-phase C18 column (Agilent ZORBAX Eclipse AAA, 4.6 × 150 mm, 5 μm). Derivatization and gradient elution were performed following standard amino acid analysis procedures, and detection was carried out at a wavelength of 338 nm [21]. All experiments were conducted in triplicate, and results are presented as mean values ± standard deviation (SD).

## 3 Results and discussion

### 3.1 Dynamic regulation of *ilvA* gene for two-stage production of 2-oxobutyric acid

**Fig. 1:**
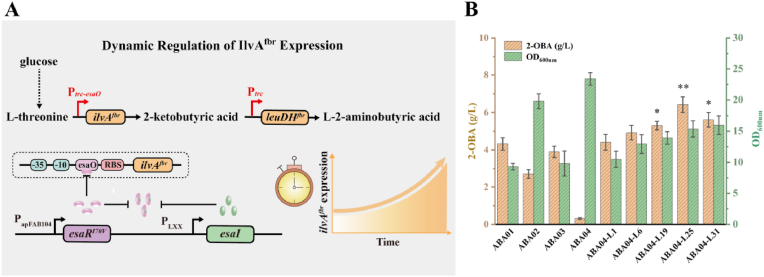
Dynamic regulation of *ilvA* expression for two-stage 2-OBA production in engineered *E. coli*. (A) Schematic diagram of the Esa quorum-sensing regulatory system used for dynamic control of *ilvA* expression. (B) Effects of promoter regulation on 2-OBA synthesis and cell growth. Data are presented as mean ± SD (n = 3). Statistical significance was evaluated using two-tailed student's *t*-test (∗∗*P* < 0.01, ∗*P* < 0.05).

In *E. coli*, 2-OBA is generated from l-threonine by threonine deaminase (IlvA). IlvA activity is subject to feedback inhibition by l-isoleucine, but this inhibition can be alleviated by targeted mutations such as F352A and R362F [22]. However, excessive accumulation of 2-OBA has been shown to inhibit both cell growth and L-2-ABA production, highlighting the need for precise temporal control of IlvA expression [23]. To achieve dynamic control over 2-OBA synthesis and minimize its inhibitory effects, separating the biosynthesis process into distinct growth and production phases could be a promising strategy to enhance L-2-ABA production. The Esa-QS circuit, which senses biomass accumulation, has been shown to effectively control circuit activation [15]. In the QS system, the EsaR protein binds to the esaO site in the P_*esaR*_ promoter to repress the expression of downstream target genes. AHL synthesized by EsaI interacts with EsaR to form the AHL-EsaR complex, which relieves repression of the P_*esaR*_ promoter (Fig. 1A). The rate of AHL synthesis is determined by the expression strength of EsaI. As cell density increases, AHL gradually accumulates, eventually leading to full activation of transcription from the P_*esaR*_ promoter.

In this study, the THR47 strain was used as the chassis cell to provide sufficient precursors for L-2-ABA biosynthesis. To relieve feedback inhibition of threonine deaminase, the IlvA^F352A, R362F^ gene driven by the P_*trc*_ and P_*esaR*_ promoters were respectively integrated into the genome of the l-threonine-producing strain THR47, yielding strains ABA01 and ABA02. Shake flask fermentation showed that ABA01 and ABA02 produced 4.32 g/L and 2.71 g/L 2-OBA, respectively (Fig. 1B). The strong P_*trc*_ promoter in ABA01 drove higher 2-OBA production but caused significant growth inhibition, whereas the low titer in ABA02 was likely due to the insufficient strength of the P_*esaR*_ promoter. To simultaneously balance cell growth and efficient 2-OBA synthesis, a hybrid promoter (P_*trc-esaO*_) derived from the Esa-QS circuit was introduced. Specifically, the EsaO binding site was inserted downstream of the transcription initiation site of the P_*trc*_ promoter [15], generating a P_*trc-esaO*_ variant for the regulation of *ilvA*^*mut*^ expression. In the absence of AHL, EsaR binding to EsaO would repress P_*trc-esaO*_ transcription. Conversely, sufficient AHL binding to EsaR would release repression of the P_*trc-esaO*_ promoter. Integrating P_*trc-esaO*_-driven *ilvA*^*mut*^ into THR47 to generate ABA03 resulted in 3.90 g/L 2-OBA. Subsequently, the *esaR*^*I70V*^ gene, constitutively expressed under the control of the apFAB104 promoter, was further overexpressed, resulting in a pronounced reduction in 2-OBA accumulation. Then, the expression library of *esaI* gene was constructed using promoters with different expression strengths (P_*L1*_ > P_*L6*_ > P_*L19*_ > P_*L25*_ > P_*L31*_) to generate ABA04-L1, ABA04-L6, ABA04-L19, ABA04-L25, and ABA04-L31 [14]. As shown in Fig. 1B, 2-OBA production first increased and then declined as EsaI expression strength decreased. The best-performing strain, ABA04-L25, achieved a titer of 6.42 g/L under the two-stage fermentation strategy, reaching an OD_600_ of 15.36, which was 64.8 % higher than that of ABA01, and exhibiting a 48.6 % improvement in 2-OBA production. This demonstrated that combining dynamic promoter regulation with the two-stage fermentation strategy effectively addresses the imbalance between cell growth and production.

**Fig. 2:**
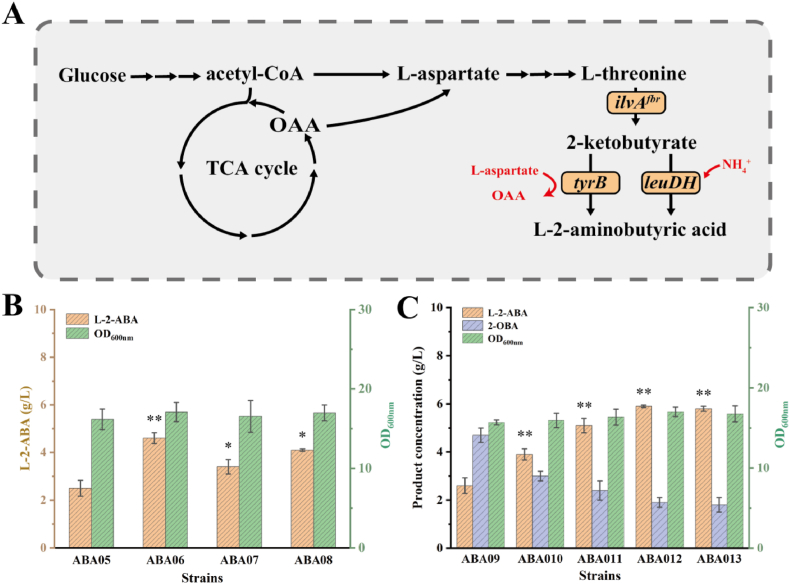
Optimization of LeuDH expression for efficient biosynthesis of L-2-ABA. (A) Schematic comparison of two routes for 2-OBA conversion to L-2-ABA. (B) Comparison of different 2-OBA-to-L-2-ABA conversion enzymes expressed in strain ABA04-L25. (C) Optimization of leuDH K72A expression through chromosomal integration and copy-number tuning. Statistical significance is indicated as ∗∗*P* < 0.01, ∗*P* < 0.05.

### 3.2 Optimizing LeuDH expression for efficient synthesis of L-2-ABA

Previously, two methods were reported for the rapid conversion of 2-OBA to L-2-ABA. The first approach employs specific amino acid transaminases, which use amino acids as amino donors to convert 2-OBA into L-2-ABA while generating by-products. For example, tyrosine aminotransferase (TyrB) can catalyze the reaction between 2-OBA and aspartate to yield L-2-ABA and oxaloacetate [24]. In threonine biosynthesis, oxaloacetate can then be recycled to l-aspartate by aspartate aminotransferase, thereby enabling regeneration of the amino donor (Fig. 2A). However, this strategy is limited by its dependence on amino acid donors and the low substrate affinity of the enzyme, resulting in reduced reaction efficiency. Another intensively investigated method is the direct reductive amination of 2-OBA by specific amino acid dehydrogenases to generate L-2-ABA. This method directly uses ammonia as the amino donor, eliminating the need for additional amino acids. Moreover, this reaction produces only water as a by-product, thereby simplifying downstream purification.

To facilitate the efficient transfer of carbon flux from 2-OBA to L-2-ABA, the biosynthetic module was optimized and validated in strain ABA04-L25. Tyrosine aminotransferase from *E. coli*, a mutant leucine dehydrogenase (LeuDH K72A) from *Exiguobacterium sibiricum* [25], and LeuDH from *Bacillus cereus* and *Thermoactinomyces intermedius* were individually expressed in ABA04-L25 using the low-copy plasmid pTH18kr [26], resulting in the construction of strains ABA05–ABA08 respectively. As a result, the L-2-ABA titers of strains ABA05, ABA06, ABA07, and ABA08 were 2.51 g/L, 4.63 g/L, 3.42 g/L, and 4.10 g/L, respectively. Given that ABA05 employed an aminotransferase pathway relying on l-aspartate as the amino donor, its comparatively low titer implied that precursor availability could constrain L-2-ABA biosynthesis. To examine this hypothesis, various concentrations of l-aspartate (1, 2, 3, 4, and 5 g/L) were supplemented in shake-flask cultures. As shown in supporting information Fig. S1, supplementation with 1–5 g/L l-aspartate had no significant effect on L-2-ABA production, with titers remaining around 2.6–3.0 g/L across all tested concentrations. These results indicated that l-aspartate availability was not a major limiting factor for L-2-ABA biosynthesis under these conditions. In contrast, strain ABA06 harboring LeuDH K72A achieved a higher titer of 4.63 g/L, indicating that the reductive amination pathway catalyzed by this mutant enzyme was more favorable for efficient L-2-ABA biosynthesis.

To improve genetic stability and reduce plasmid burden, LeuDH K72A from *Exiguobacterium sibiricum* was integrated into the genome of ABA04-L25, and strain ABA09 was thereby generated. In shake-flask fermentation, ABA09 produced 2.63 g/L L-2-ABA while 4.71 g/L 2-OBA was accumulated (Fig. 2C). To optimize the expression level of leuDH K72A, additional genomic copies were introduced, resulting in strains ABA10 to ABA13 containing two to five copies, respectively. Under these conditions, L-2-ABA titers of 3.91, 5.10, 5.93, and 5.81 g/L were obtained for ABA10–ABA13, respectively, showing a copy-number–dependent increase that reached a maximum at four copies. Meanwhile, the accumulation of 2-OBA gradually decreased with increasing leuDH K72A expression; for instance, ABA12 accumulated only 1.90 g/L 2-OBA (Fig. 2C). No additional improvement was observed when the copy number was further increased to five, as the titer of ABA13 was comparable to that of ABA12. These results suggest that leuDH K72A was no longer the rate-limiting factor under these conditions. Notably, as 2-OBA accumulation was reduced with increasing leuDH K72A expression, biomass was partially restored across the engineered strains, indicating that alleviation of 2-OBA toxicity contributed to improved cell growth.

### 3.3 *In silico* prediction of metabolic strategies for enhancing L-2-ABA biosynthesis

**Fig. 3:**
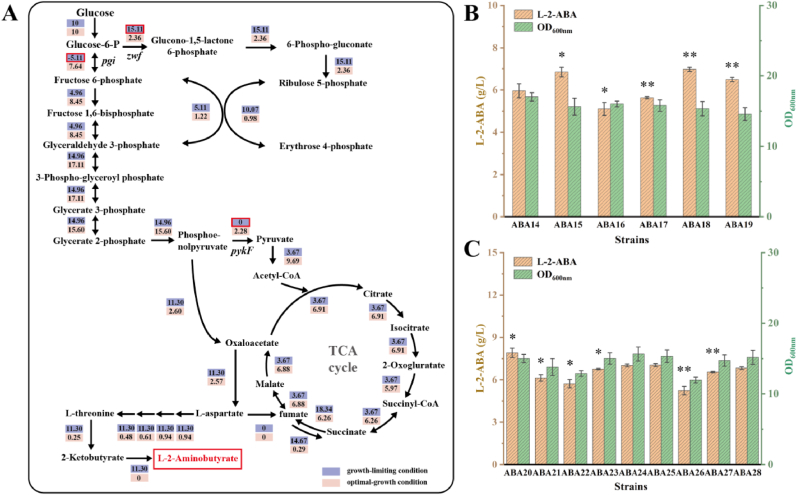
Genome-scale metabolic modeling–guided prediction and experimental validation of metabolic strategies for enhancing L-2-ABA biosynthesis. (A) FBA–based simulation of intracellular carbon flux distribution in the *E. coli* iML1515 model under both maximal-growth and growth-limited conditions. (B) Experimental validation of PPP enhancement through *pgi* attenuation and *zwf* overexpression. (C) Model-guided identification and experimental verification of gene knockout targets predicted by OptKnock and OptGene algorithms. Statistical significance is indicated as ∗∗*P* < 0.01, ∗*P* < 0.05.

To further improve L-2-ABA production, potential metabolic engineering targets were predicted using genome-scale metabolic network modeling (GSMN). Since the chassis strain THR47 used for constructing the L-2-ABA-producing strain was derived from *E. coli* K-12 MG1655, the iML1515 model reconstructed from this parental strain was adopted as the base framework for subsequent model modifications [27]. As the native pathway from 2-OBA to L-2-ABA is absent in *E. coli*, the LeuDH-catalyzed reaction (LEUDH_2abut: 2obut_c + nh4_c + nadh_c -> 2abut_c + nad_c + h2o_c) was introduced into the iML1515 model background. Subsequently, FBA was performed to simulate the intracellular carbon flux distributions under two scenarios: (i) maximal growth, and (ii) growth-limited conditions (biomass fixed at 0.1) with maximized L-2-ABA production. The resulting changes in carbon fluxes through central metabolism and the L-2-ABA synthetic pathways were visualized using a flux map (Fig. 3A).

In the FBA simulations, the glucose uptake rate was fixed at 10 mmol·gDW^−1^·h^−1^, and the flux of phosphoglucose isomerase (PGI), encoded by the *pgi* gene, was predicted to be −5.11 mmol·gDW^−1^·h^−1^. This negative flux reflects a model-predicted metabolic redistribution trend, namely reduced flux through the EMP pathway and increased carbon flux toward the pentose phosphate pathway (PPP), which may be beneficial for L-2-ABA biosynthesis. Although PGI is a reversible enzyme, under physiological conditions it is thermodynamically more favorable for the glycolytic conversion of glucose-6-phosphate to fructose-6-phosphate. Therefore, the reverse synthesis of glucose-6-phosphate from fructose-6-phosphate, as predicted by the model, is thermodynamically unfavorable and unlikely to occur efficiently *in vivo*. Nevertheless, the simulation suggested that decreasing the flux into the EMP pathway while enhancing the flux through the pentose phosphate pathway may lead to an improvement in L-2-ABA synthesis. To promote PPP flux, the expression of *pgi* was attenuated and *zwf*, encoding glucose-6-phosphate dehydrogenase as the first enzyme of the PPP, was overexpressed. Specifically, strain ABA14 was constructed by replacing the start codon of *pgi* with GTG, while strains ABA15–ABA19 were generated by substituting the native promoter of *zwf* with a set of promoters of different strengths (J23100, J23110, J23118, J23119, and P_*trc*_). The effects of these genetic modifications on L-2-ABA production were subsequently assessed in shake-flask fermentation. Compared with the strain ABA12, ABA14 did not exhibit a significant change in L-2-ABA production, indicating that attenuation of *pgi* alone was not sufficient to redirect carbon flux toward the PPP. Accordingly, strains ABA15–ABA19, in which *zwf* expression was further optimized, redirected more carbon flux into this pathway, with ABA18 (*zwf* driven by the J23119 promoter) achieving the highest titer of 6.94 g/L (Fig. 3B).

While experimental validation of PPP enhancement demonstrated the feasibility of rational flux redirection, the resulting improvements in L-2-ABA production were relatively limited. To identify additional metabolic engineering targets, two computational approaches were subsequently employed to predict potential gene knockouts to further enhance L-2-ABA biosynthesis. The first approach, OptKnock, is a bilevel linear programming–based optimization framework that identifies gene knockout combinations by coupling product formation with cell growth through simultaneous optimization of biomass and target synthesis [28]. The second approach, OptGene, is based on a genetic algorithm that explores a large solution space by mimicking evolutionary processes such as selection, crossover, and mutation [29]. When the specific growth rate was constrained to a low level (0.1 h^−1^) to mimic growth-limited production states, both methods predicted five candidate engineering targets. OptKnock predicted *pykF* (ABA20), *umpG* (ABA21), *waaZ* (ABA22), *phnN* (ABA23), and *nudL* (ABA24), whereas OptGene predicted *pykF*, *astB* (ABA25), *thiM* (ABA26), *phoE* (ABA27), and *guaD* (ABA28). After merging the results and removing duplicates, a total of nine distinct candidate genes were obtained. These predictions were subsequently validated through single-gene knockout experiments (Fig. 3C). Among them, the *pykF*-deficient strain exhibited the most pronounced improvement in L-2-ABA production, reaching a titer of 7.91 g/L, which was 13.5 % higher than that of ABA19. This outcome was consistent with the model prediction, indicating that *pykF* plays a critical role in the conversion of phosphoenolpyruvate (PEP) to pyruvate in central metabolism; its deletion enhanced PEP availability and consequently promoted carbon flux toward the L-2-ABA biosynthetic pathway.

### 3.4 Engineering NADH supply to improve L-2-ABA production

Redox imbalance impairs cell growth and product synthesis [30,31]. Cofactor engineering therefore represents a critical strategy in metabolic engineering to address such limitations [18,32]. In particular, the reducing power of NADH is essential for the rapid conversion of 2-OBA to L-2-ABA catalyzed by LeuDH K72A. A variety of strategies have been explored to increase intracellular NADH availability, including modulation of endogenous metabolic pathways and introduction of heterologous cofactor regeneration systems [33]. For instance, formate dehydrogenase (FDH) generates NADH by oxidizing formate to CO_2_ and H_2_O, a process that avoids the formation of undesired byproducts [34]. The advantage of this strategy is that it does not result in the production of additional byproducts.

**Fig. 4:**
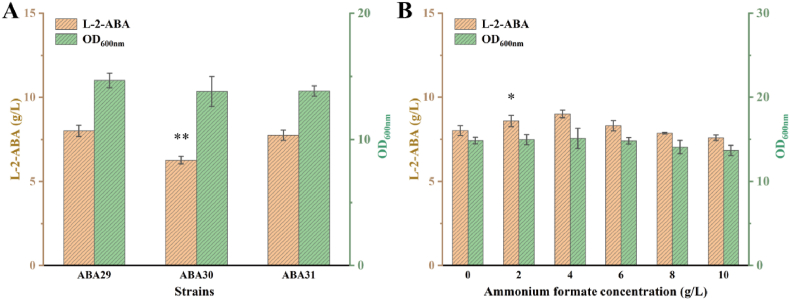
Engineering NADH regeneration pathways to enhance L-2-ABA biosynthesis. (A) Effects of cofactor supply regulation on L-2-ABA biosynthesis. (B) Effects of ammonium formate supplementation on L-2-ABA production. Statistical significance is indicated as ∗∗*P* < 0.01, ∗*P* < 0.05.

Based on these considerations, FDH from *Candida boidinii* was first introduced into strain ABA20 to generate ABA29, aiming to enhance the effective intracellular NADH supply. However, the L-2-ABA titer of ABA29 increased only slightly to 8.02 g/L, likely due to limited formate availability that restricted NADH regeneration. To test this hypothesis, pyruvate formate lyase (PFL, encoded by *pflB*) was overexpressed in ABA29 to generate ABA30, thereby increasing the flux from pyruvate to formate (Fig. S2). Unexpectedly, overexpression of *pflB* reduced the L-2-ABA titer to 6.27 g/L (Fig. 4A), possibly because carbon flux was diverted toward formate synthesis, thereby decreasing L-2-ABA production. These results indicated that increasing endogenous formate supply was not an effective strategy for NADH regeneration. Given that enhancing endogenous formate synthesis proved ineffective for improving NADH regeneration, exogenous supplementation with ammonium formate was subsequently tested at concentrations of 2, 4, 6, 8, and 10 g/L. Supplementation with 2 g/L and 4 g/L ammonium formate increased L-2-ABA titers by 7.11 % and 12.30 %, respectively. A modest improvement of 3.61 % was observed at 6 g/L, whereas higher concentrations (8 g/L and 10 g/L) resulted in decreases of 1.93 % and 5.32 %, respectively (Fig. 4B). These results suggest that moderate ammonium formate supplementation enhances NADH regeneration and thereby promotes L-2-ABA production, whereas excessive supplementation exerts inhibitory effects. Therefore, optimization of ammonium formate feeding strategies will be essential for future fermentation processes. In addition to external supplementation, *E. coli* possesses an intrinsic cofactor-balancing mechanism that regulates the intracellular NADPH/NADH ratio. Among the related enzymes, pyridine nucleotide transhydrogenase (encoded by *sthA*) catalyzes the conversion of NADPH to NADH. To exploit this mechanism, *sthA* expression was further enhanced, generating strain ABA31. As a result, overexpression of *sthA* decreased the L-2-ABA titer by 3.20 % (Fig. 4A), which was likely due to reduced NADPH availability interfering with the upstream l-threonine biosynthetic pathway, thereby limiting L-2-ABA formation.

### 3.5 Balanced regulation of l-threonine supply and 2-OBA catabolism to enhance L-2-ABA biosynthesis

**Fig. 5:**
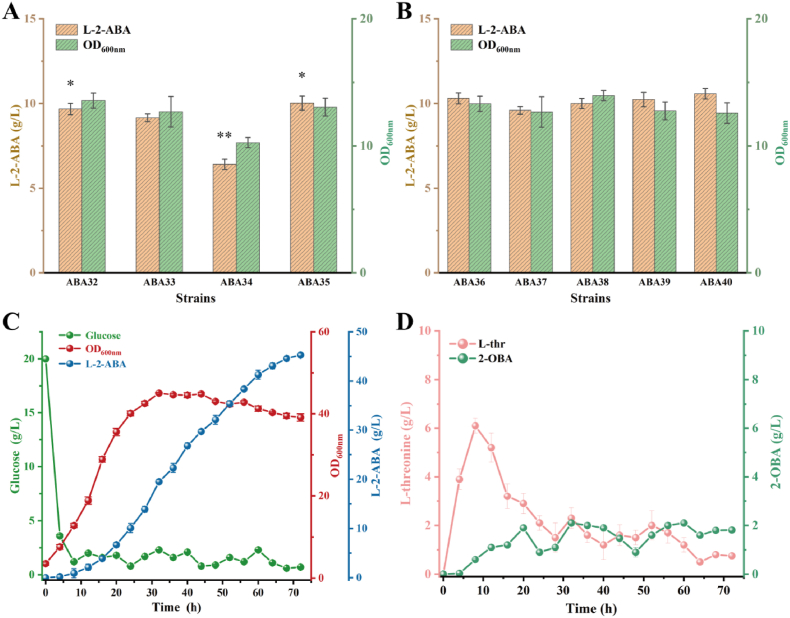
Balanced precursor regulation and transcriptional optimization enhance L-2-ABA biosynthesis. (A) Effects of *rhtA* and *rhtC* deletions on L-2-ABA production. (B) Effects of transcriptional regulators on L-2-ABA biosynthesis. (C) Fed-batch fermentation parameters of ABA40 in a 5 L bioreactor. (D) Accumulation of by-products in the fermentation broth. Statistical significance is indicated as ∗∗*P* < 0.01, ∗*P* < 0.05.

In previous studies, in order to construct the l-threonine overproducing strain THR47, the l-threonine efflux system in the chassis strain was enhanced to reduce intracellular accumulation [35]. However, a low intracellular concentration of l-threonine was found to be unfavorable for L-2-ABA biosynthesis [36]. Therefore, rational regulation of the efflux system is required to maintain an adequate intracellular supply of l-threonine. The efflux proteins RhtA and RhtC, known to mediate l-threonine export, were deleted individually and in combination. Of these, strain ABA32 (Δ*rhtA*), ABA33 (Δ*rhtC*), and ABA34 (Δ*rhtA*, Δ*rhtC*) accumulated 9.68, 9.16, and 6.42 g/L of L-2-ABA, respectively (Fig. 5A). These results indicated that moderate attenuation of l-threonine efflux helped maintain an optimal intracellular balance of the precursor, thereby promoting L-2-ABA biosynthesis. In contrast, complete blockage of the l-threonine export system inhibited cell growth and reduced product formation, as excessive intracellular l-threonine accumulation likely caused overproduction of 2-OBA, leading to metabolic stress and feedback inhibition of upstream biosynthetic enzymes. Therefore, balanced regulation of l-threonine transport was crucial for maintaining metabolic homeostasis and achieving high-level L-2-ABA production.

In addition, acetohydroxy acid synthase (AHAS, encoded by the *ilvIH* genes) in *E. coli* has a high affinity for 2-OBA, which can lead to excessive consumption of this precursor [19]. To mitigate 2-OBA depletion, the expression of *ilvIH* was not deleted but instead placed under the control of the growth-associated promoter P_*fliC*_. The transcriptional activity of P_*fliC*_ is growth-phase dependent, with relatively high activity during the pre-logarithmic and mid-logarithmic phases, followed by a marked decrease in the late logarithmic phase [37]. By replacing the native promoter of *ilvIH* with P_*fliC*_, strain ABA35 achieved an L-2-ABA titer of 10.03 g/L, demonstrating that dynamic regulation of precursor catabolism is an effective strategy for improving L-2-ABA production.

### 3.6 Engineering transcriptional regulators to improve strain resistance

The efficient synthesis of chemicals depends not only on pathway engineering but is also strongly influenced by carbon flux distribution, redox balance, and stress tolerance [38,39]. Under high-production conditions, accumulation of intermediates or target products often induces metabolic stress, leading to growth retardation and reduced production efficiency [40]. Therefore, strategies such as reprogramming carbon and nitrogen allocation, optimizing redox homeostasis, and strengthening metabolic tolerance are expected to further enhance L-2-ABA production. In this context, transcriptional regulators, which enable global control of multi-gene networks, represent an effective strategy for cell-level optimization [41]. For example, PdhR represses the transcription of the pyruvate dehydrogenase complex and respiratory chain components, thereby influencing the allocation of pyruvate between energy metabolism and precursor biosynthesis [19]. SpoT mediates the stringent response through (p)ppGpp signaling, and introduction of the SpoT^R290E, K292D^ variant has been reported to increase the intracellular pyruvate pool and improve production [42]. Furthermore, the heterologous regulator IrrE from *Deinococcus radiodurans* has been shown to enhance multiple stress tolerances in *E. coli*, thereby contributing to improved metabolic robustness [43].

To assess the impact of transcriptional regulators on L-2-ABA biosynthesis, the genes *pdhR*, *rpoS*, and *irrE* were individually integrated into the engineered strain ABA35, generating strains ABA36–ABA38, respectively. In addition, the SpoT^R290E, K292D^ mutant was used to replace the wild-type *spoT* gene, yielding strain ABA39. The fermentation results showed that overexpression of PdhR (ABA36) and replacement of the wild-type *spoT* with the SpoT^R290E, K292D^ mutant (ABA39) increased the L-2-ABA titer by 2.8 % and 2.1 %, respectively, compared with strain ABA35 (Fig. 5B). These improvements are consistent with the regulatory role of PdhR in reducing excessive pyruvate flux into the TCA cycle and of SpoT^R290E, K292D^ in enlarging the intracellular pyruvate pool, thereby enhancing precursor availability. Based on these insights, pdhR was further integrated into the genome of ABA39 to construct the combined regulatory strain ABA40, resulting in an L-2-ABA titer of 10.58 g/L. In contrast, overexpression of RpoS (ABA37) decreased the L-2-ABA titer by 4.3 %, likely due to stress-induced transcriptional reprogramming that diverted cellular resources away from product formation. Meanwhile, engineering of IrrE (ABA38) did not lead to significant changes in L-2-ABA production under the tested conditions, suggesting that their regulatory effects may be condition-dependent or require more complex network rewiring to manifest. Collectively, these findings highlight that precise modulation of transcriptional regulators can influence the balance between central metabolism, stress response, and precursor allocation, thereby providing useful insights for further optimization of L-2-ABA biosynthesis.

To evaluate the fermentation performance of strain ABA40 for L-2-ABA production, a fed-batch process was carried out in a 5 L bioreactor. As shown in Fig. 5C, after 72 h, strain ABA40 produced 45.3 g/L L-2-ABA, corresponding to a yield of 0.31 g/g glucose. At the end of fermentation, 0.75 g/L threonine and 1.81 g/L 2-OBA accumulated in the supernatant (Fig. 5D). These results suggest that further improvements should focus on enhancing the conversion of 2-OBA to L-2-ABA. In conclusion, strain ABA40 was successfully engineered for the *de novo* synthesis of L-2-ABA. Notably, the fermentation process did not require antibiotics or inducers to maintain plasmid stability and gene expression. In addition, optimization of inoculum size, medium composition, and substrate feeding rate may further enhance L-2-ABA production. Overall, this work provides valuable insights into the development of cost-effective and robust microbial platforms for the industrial production of amino acid derivatives.

## 4 Conclusion

In this study, a reprogrammed *Escherichia coli* ABA40 was constructed to enable *de novo* synthesis of L-2-ABA. Dynamic regulation of *ilvA* via a quorum-sensing-controlled P_*trc-esaO*_ circuit enabled temporal separation of growth and production phases, effectively balancing biomass accumulation and 2-OBA generation. Integration and multi-copy optimization of mutant LeuDH (K72A) from *Exiguobacterium sibiricum* markedly improved the conversion efficiency of 2-OBA to L-2-ABA. Guided by genome-scale metabolic modeling, the deletion of *pykF* redirected phosphoenolpyruvate flux toward product synthesis, resulting in a 13.5 % increase in titer. Redox engineering through heterologous formate dehydrogenase expression and moderate ammonium formate supplementation enhanced NADH regeneration, highlighting the importance of maintaining cofactor balance for efficient L-2-ABA synthesis. Balanced regulation of l-threonine efflux and dynamic control of *ilvIH* expression effectively prevented excessive precursor depletion. Fine-tuning of transcriptional regulators PdhR and SpoT^R290E,K292D^ enhanced pyruvate allocation, stress tolerance, and carbon utilization efficiency. The engineered strain ABA40 achieved 45.3 g/L of L-2-ABA with a yield of 0.31 g/g glucose in 72 h fed-batch fermentation without antibiotics or inducers.

## CRediT authorship contribution statement

**Zhenqiang Zhao:** Writing – original draft, Visualization, Investigation, Funding acquisition. **Yizheng Liu:** Visualization, Investigation. **Rongshuai Zhu:** Investigation. **Fengyu Yang:** Visualization. **Zhifei Liu:** Investigation. **Jiajia You:** Visualization, Methodology. **Xuewei Pan:** Visualization, Methodology. **Jianming Yang:** Methodology. **Zhiming Rao:** Visualization, Funding acquisition, Conceptualization.

## Data availability Statement

The authors declare that all data in the results of this study are available in the paper (and its supplementary document), or from the corresponding authors upon request.

## Declaration of competing interest

The authors declare that they have no known competing financial interests or personal relationships that could have appeared to influence the work reported in this paper.

## References

[1] Shin J.-S., Kim B.-G. (2009). Transaminase-catalyzed asymmetric synthesis of L-2-aminobutyric acid from achiral reactants. Biotechnol Lett.

[2] Zhang K., Li H., Cho K.M., Liao J.C. (2010). Expanding metabolism for total biosynthesis of the nonnatural amino acid L-homoalanine. Proc Natl Acad Sci.

[3] Tang X.L., Lu X.F., Wu Z.M., Zheng R.C., Zheng Y.G. (2018). Biocatalytic production of (S)-2-aminobutanamide by a novel d-aminopeptidase from *Brucella sp.* with high activity and enantioselectivity. J Biotechnol.

[4] Mathew S., Bea H., Nadarajan S.P., Chung T., Yun H. (2015). Production of chiral β-amino acids using ω-transaminase from *Burkholderia graminis*. J Biotechnol.

[5] Zhang L., Hong Y., Lu J., Wang Y., Luo W. (2024). Semi-rational engineering of omega-transaminase for enhanced enzymatic activity to 2-ketobutyrate. Enzym Microb Technol.

[6] Zhu L., Tao R., Wang Y., Jiang Y., Lin X., Yang Y. (2011). Removal of L-alanine from the production of L-2-aminobutyric acid by introduction of alanine racemase and d-amino acid oxidase. Appl Microbiol Biotechnol.

[7] Galkin A., Kulakova L., Yoshimura T., Soda K., Esaki N. (1997). Synthesis of optically active amino acids from alpha-keto acids with *Escherichia coli* cells expressing heterologous genes. Appl Environ Microbiol.

[8] Tao R., Jiang Y., Zhu F., Yang S. (2014). A one-pot system for production of L-2-aminobutyric acid from L-threonine by L-threonine deaminase and a NADH-regeneration system based on L-leucine dehydrogenase and formate dehydrogenase. Biotechnol Lett.

[9] Zhu L., Wu Z., Jin J.-M., Tang S.-Y. (2016). Directed evolution of leucine dehydrogenase for improved efficiency of l-tert-leucine synthesis. Appl Microbiol Biotechnol.

[10] Dedeakayoğulları H., Valjakka J., Turunen O., Yilmazer B., Demir Ğ., Jänis J. (2023). Application of reductive amination by heterologously expressed *Thermomicrobium roseum* L-alanine dehydrogenase to synthesize L-alanine derivatives. Enzym Microb Technol.

[11] Hu Y., Xu G., Ni Y. (2024). A novel and thermostable phenylalanine dehydrogenase for efficient synthesis of bulky aromatic amino acids and derivatives. Mol Catal.

[12] Luo W., Zhu J., Zhao Y., Zhang H., Yang X., Liu Y. (2020). Cloning and expression of a novel leucine dehydrogenase: characterization and L-tert-leucine production. Front Bioeng Biotechnol.

[13] Ge C., Yu Z., Sheng H., Shen X., Sun X., Zhang Y. (2022). Redesigning regulatory components of quorum-sensing system for diverse metabolic control. Nat Commun.

[14] Gupta A., Reizman I.M., Reisch C.R., Prather K.L. (2017). Dynamic regulation of metabolic flux in engineered bacteria using a pathway-independent quorum-sensing circuit. Nat Biotechnol.

[15] Dinh C.V., Prather K.L.J. (2019). Development of an autonomous and bifunctional quorum-sensing circuit for metabolic flux control in engineered *Escherichia coli*. Proc Natl Acad Sci U S A.

[16] Zhao Z., Zhu R., Shi X., Yang F., Xu M., Shao M. (2025). Combining biosensor and metabolic network optimization strategies for enhanced L-threonine production in *Escherichia coli*. Biotechnol Biofuels Bioprod.

[17] Kim S., Koh S., Kang W., Yang J.K. (2022). The Crystal structure of L-leucine dehydrogenase from *Pseudomonas aeruginosa*. Mol Cells.

[18] Wang M., Chen B., Fang Y., Tan T. (2017). Cofactor engineering for more efficient production of chemicals and biofuels. Biotechnol Adv.

[19] Hao Y., Pan X., Xing R., You J., Hu M., Liu Z. (2022). High-level production of L-valine in *Escherichia coli* using multi-modular engineering. Bioresour Technol.

[20] Xu J.Z., Ruan H.Z., Chen X.L., Zhang F., Zhang W. (2019). Equilibrium of the intracellular redox state for improving cell growth and L-lysine yield of *Corynebacterium glutamicum* by optimal cofactor swapping. Microb Cell Fact.

[21] Wang Y., Yao C., Huang D., Li H., Li Y., Liu Z. (2024). Metabolic engineering of *Corynebacterium glutamicum* CGY-PG-304 for promoting gamma-aminobutyric acid production. Syst Microbiol Biomanuf.

[22] Chen L., Chen Z., Zheng P., Sun J., Zeng A.P. (2013). Study and reengineering of the binding sites and allosteric regulation of biosynthetic threonine deaminase by isoleucine and valine in *Escherichia coli*. Appl Microbiol Biotechnol.

[23] Zhang H., Song F., Wang K., Wu F., Deng L., Qiu K. (2026). Precise L-threonine-to-L-isoleucine pathway regulation for engineering high-efficiency whole-cell biocatalysts. Synth Syst Biotechnol.

[24] Fotheringham I.G., Grinter N., Pantaleone D.P., Senkpeil R.F., Taylor P.P. (1999). Engineering of a novel biochemical pathway for the biosynthesis of L-2-aminobutyric acid in *Escherichia coli* K12. Bioorg Med Chem.

[25] Chen J., Zhu R., Zhou J., Yang T., Zhang X., Xu M. (2021). Efficient single whole-cell biotransformation for L-2-aminobutyric acid production through engineering of leucine dehydrogenase combined with expression regulation. Bioresour Technol.

[26] Tao R., Jiang Y., Zhu F., Yang S. (2014). A one-pot system for production of L-2-aminobutyric acid from L-threonine by L-threonine deaminase and a NADH-regeneration system based on L-leucine dehydrogenase and formate dehydrogenase. Biotechnol Lett.

[27] Monk J.M., Lloyd C.J., Brunk E., Mih N., Sastry A., King Z. (2017). iML1515, a knowledgebase that computes *Escherichia coli* traits. Nat Biotechnol.

[28] Burgard A.P., Pharkya P., Maranas C.D. (2003). Optknock: a bilevel programming framework for identifying gene knockout strategies for microbial strain optimization. Biotechnol Bioeng.

[29] Patil K.R., Rocha I., Forster J., Nielsen J. (2005). Evolutionary programming as a platform for *in silico* metabolic engineering. BMC Bioinf.

[30] Lee J.W., Kim H.U., Choi S., Yi J., Lee S.Y. (2011). Microbial production of building block chemicals and polymers. Curr Opin Biotechnol.

[31] Xu J., Tao Y., Shan Q., Feng Y., Wang Y., Liu Z. (2025). Optimized biosynthetic pathway for nonnatural amino acids: an efficient approach for L-2-aminobutyric acid production. Biotechnol Bioeng.

[32] Ma Q., Zhang Q., Xu Q., Zhang C., Li Y., Fan X. (2017). Systems metabolic engineering strategies for the production of amino acids. Synth Syst Biotechnol.

[33] Wang Y., San K.Y., Bennett G.N. (2013). Cofactor engineering for advancing chemical biotechnology. Curr Opin Biotechnol.

[34] Tan W.T. (2013). Improved 1,3-propanediol production by engineering the 2,3-butanediol and formic acid pathways in integrative recombinant *Klebsiella pneumoniae*. J Biotechnol.

[35] Zhao Z., You J., Shi X., Cai M., Zhu R., Yang F. (2025). Multi-module engineering to guide the development of an efficient L-threonine-producing cell factory. Bioresour Technol.

[36] Xu J.-M., Li J.-Q., Zhang B., Liu Z.-Q., Zheng Y.-G. (2019). Fermentative production of the unnatural amino acid L-2-aminobutyric acid based on metabolic engineering. Microb Cell Fact.

[37] Cai M., Zhao Z., Li X., Xu Y., Xu M., Rao Z. (2022). Development of a nonauxotrophic L-homoserine hyperproducer in *Escherichia coli* by systems metabolic engineering. Metab Eng.

[38] Lancaster Louis, Abdallah Walaa, Banta Scott (2018). Engineering enzyme microenvironments for enhanced biocatalysis. Chem Soc Rev.

[39] Zhang L., Li Y., Xiao F., Zhang Y., Zhang L., Ding Z. (2024). Transcriptional modulation of the global regulator CodY using a conditional CRISPRi system in *Bacillus licheniformis*. Syst Microbiol Biomanuf.

[40] Wang J., Zhang Y., Chen Y., Lin M., Lin Z. (2012). Global regulator engineering significantly improved *Escherichia coli* tolerances toward inhibitors of lignocellulosic hydrolysates. Biotechnol Bioeng.

[41] Tang M., Pan X., Yang T., You J., Zhu R., Yang T. (2023). Multidimensional engineering of *Escherichia coli* for efficient synthesis of L-tryptophan. Bioresour Technol.

[42] Hao Y., Ma Q., Liu X., Fan X., Men J., Wu H. (2020). High-yield production of L-valine in engineered *Escherichia coli* by a novel two-stage fermentation. Metab Eng.

[43] Zhao P., Zhou Z., Zhang W., Lin M., Chen M., Wei G. (2015). Global transcriptional analysis of *Escherichia coli* expressing IrrE, a regulator from *Deinococcus radiodurans*, in response to NaCl shock. Mol Biosyst.

