# Supplementary material for: De novo synthesis of L-2-aminobutyric acid in Escherichia coli based on multi-layered metabolic engineering strategies
Source: Synth Syst Biotechnol. 2026 Jan 15;12:374–82. doi: 10.1016/j.synbio.2026.01.004 (PMC12830291; doi:10.1016/j.synbio.2026.01.004)
Supplement: Multimedia component 1 [file mmc1.docx]

**Supplementary Table S1.**

Strains and plasmids used in this study.

| **Strain** | **Description** | **Sources** |
| --- | --- | --- |
| *E. coli* THR47 | L-threonine–producing strain; chassis strain used for L-2-ABA biosynthesis | Laboratory stock |
| *E. coli* DH5α | Cloning host | Laboratory stock |
| ABA01 | *E. coli* THR47 harboring genomically integrated P*_trc_*-driven *ilvA*^F352A, R362F^ | This work |
| ABA02 | *E. coli* THR47 harboring genomically integrated P*_esaR_*-driven *ilvA*^F352A, R362F^ | This work |
| ABA03 | *E. coli* THR47 harboring genomically integrated P*_trc-esaO_*-driven *ilvA*^F352A, R362F^ | This work |
| ABA04 | ABA03 harboring genomically integrated apFAB104 promoter-driven *esaR*^I70V^ | This work |
| ABA04-L1 | ABA04 harboring genomically integrated P*_L1_* promoter-driven *esaI* | This work |
| ABA04-L6 | ABA04 harboring genomically integrated P*_L6_* promoter-driven *esaI* | This work |
| ABA04-L19 | ABA04 harboring genomically integrated P*_L19_* promoter-driven *esaI* | This work |
| ABA04-L25 | ABA04 harboring genomically integrated P*_L25_* promoter-driven *esaI* | This work |
| ABA04-L31 | ABA04 harboring genomically integrated P*_L31_* promoter-driven *esaI* | This work |
| ABA05 | ABA04-L25 harboring plasmid pTh18kr-*tyrB* under the control of the P*_trc_* promoter | This work |
| ABA06 | ABA04-L25 harboring plasmid pTh18kr-*EsleuDH*^K72A^ under the control of the P*_trc_* promoter | This work |
| ABA07 | ABA04-L25 harboring plasmid pTh18kr-*BcleuDH* under the control of the P*_trc_* promoter | This work |
| ABA08 | ABA04-L25 harboring plasmid pTh18kr-*TileuDH* under the control of the P*_trc_* promoter | This work |
| ABA09 | ABA04-L25 harboring genomically integrated P*_trc-_*-driven *EsleuDH*^K72A^ | This work |
| ABA10 | ABA09 harboring genomically integrated P*_trc-_*-driven *EsleuDH*^K72A^ | This work |
| ABA11 | ABA10 harboring genomically integrated P*_trc-_*-driven *EsleuDH*^K72A^ | This work |
| ABA12 | ABA11 harboring genomically integrated P*_trc-_*-driven *EsleuDH*^K72A^ | This work |
| ABA13 | ABA12 harboring genomically integrated P*_trc-_*-driven *EsleuDH*^K72A^ | This work |
| ABA14 | ABA13 harboring a GTG substitution at the start codon of *pgi* | This work |
| ABA15 | ABA14 harboring the native promoter of *zwf* replaced with the J23100 promoter | This work |
| ABA16 | ABA14 harboring the native promoter of *zwf* replaced with the J23110 promoter | This work |
| ABA17 | ABA14 harboring the native promoter of *zwf* replaced with the J23118 promoter | This work |
| ABA18 | ABA14 harboring the native promoter of *zwf* replaced with the J23119 promoter | This work |
| ABA19 | ABA14 harboring the native promoter of *zwf* replaced with the P*_trc_* promoter | This work |
| ABA20 | ABA18 with *pykF* deleted | This work |
| ABA21 | ABA18 with *umpG* deleted | This work |
| ABA22 | ABA18 with *waaZ* deleted | This work |
| ABA23 | ABA18 with *phnN* deleted | This work |
| ABA24 | ABA18 with *nudL* deleted | This work |
| ABA25 | ABA18 with *astB* deleted | This work |
| ABA26 | ABA18 with *thiM* deleted | This work |
| ABA27 | ABA18 with *phoE* deleted | This work |
| ABA28 | ABA18 with *guaD* deleted | This work |
| ABA29 | ABA20 harboring genomically integrated P*_trc-_*-driven *Cbfdh* | This work |
| ABA30 | ABA29 harboring genomically integrated P*_trc-_*-driven *pflB* | This work |
| ABA31 | ABA29 harboring genomically integrated P*_trc-_*-driven *sthA* | This work |
| ABA32 | ABA29 with *rhtA* deleted | This work |
| ABA33 | ABA29 with *rhtC* deleted | This work |
| ABA34 | ABA29 with *rhtA* and *rhtC* deleted | This work |
| ABA35 | ABA32 with the native promoter of *ilvIH* replaced with the P*_fliC_* promoter | This work |
| ABA36 | ABA35 harboring genomically integrated P*_trc-_*-driven *pdhR* | This work |
| ABA37 | ABA35 harboring genomically integrated P*_trc-_*-driven *rpoS* | This work |
| ABA38 | ABA35 harboring genomically integrated P*_trc-_*-driven *irrE* | This work |
| ABA39 | ABA35 with the wild-type *spoT* replaced by *spoT*^R290E, K292D^ | This work |
| ABA40 | ABA39 harboring genomically integrated P*_trc-_*-driven PdhR | This work |
| **Plasmid** | **Description** | **Sources** |
| pTH18kr | Low-copy expression plasmid, kanamycin resistance gene, and pSC101 origin | Laboratory stock |
| pRedCas9 | CRISPR-Cas9 system, carrying Cas9 protein and λ Red system, temperature sensitive, Spe^R^ | Laboratory stock |
| pGRB | CRISPR-Cas9 system, for expression of *sg*RNA in *E. coli*, Amp^R^ | Laboratory stock |

**Supplementary Table S2.**

Primers used for real-time PCR in this study.

| Primers | Sequence (5’-3’) |
| --- | --- |
| RT-16sRNA-F | GGTGTAGCGGTGAAATGCGTAG |
| RT-16sRNA-R | CTCAAGGGCACAACCTCCAAG |
| RT-*leuDH*-F | GTGGCCGACATGGACTACATC |
| RT-*leuDH*-R | CATACCGCGGTACACGCC |

**Supplementary Fig. S1.**
Effect of L-aspartate supplementation on L-2-ABA production in strain ABA05. Statistical significance was evaluated using two-tailed Student’s *t*-test (**P* < 0.05).


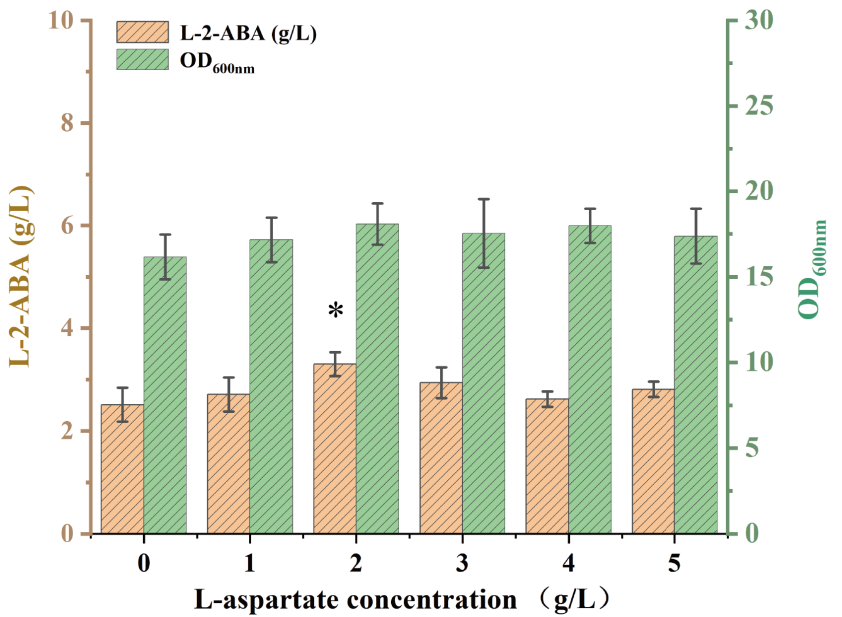


**Supplementary Fig. S2**

Effects of formate metabolism and cofactor engineering on intracellular redox balance. (A) Extracellular formate accumulation in strains ABA29 and ABA30. (B) Intracellular NADH/NADPH ratios in strains ABA29, ABA29 supplemented with 4 g/L ammonium formate (4 g/L AF), ABA30 (*pflB* overexpression), and ABA31 (*sthA* overexpression). Data are presented as mean ± SD (n = 3). Statistical significance was evaluated using two-tailed Student’s *t*-test (***P* < 0.01).

**
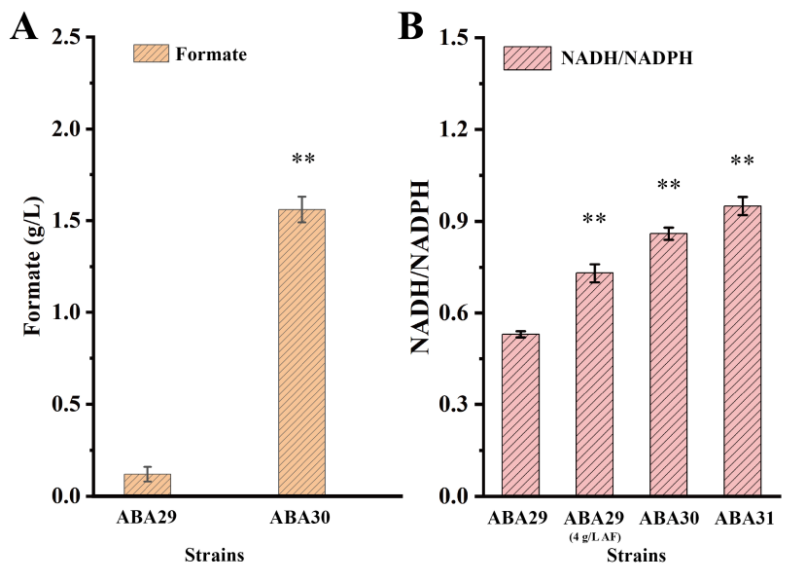
**

**Supplementary Fig. S3**

Relative transcription levels of *leuDH*^K72A^ in strains carrying different chromosomal copy numbers. Data are presented as mean ± SD (n = 3). Statistical significance was evaluated using two-tailed Student’s *t*-test (***P* < 0.01).

**
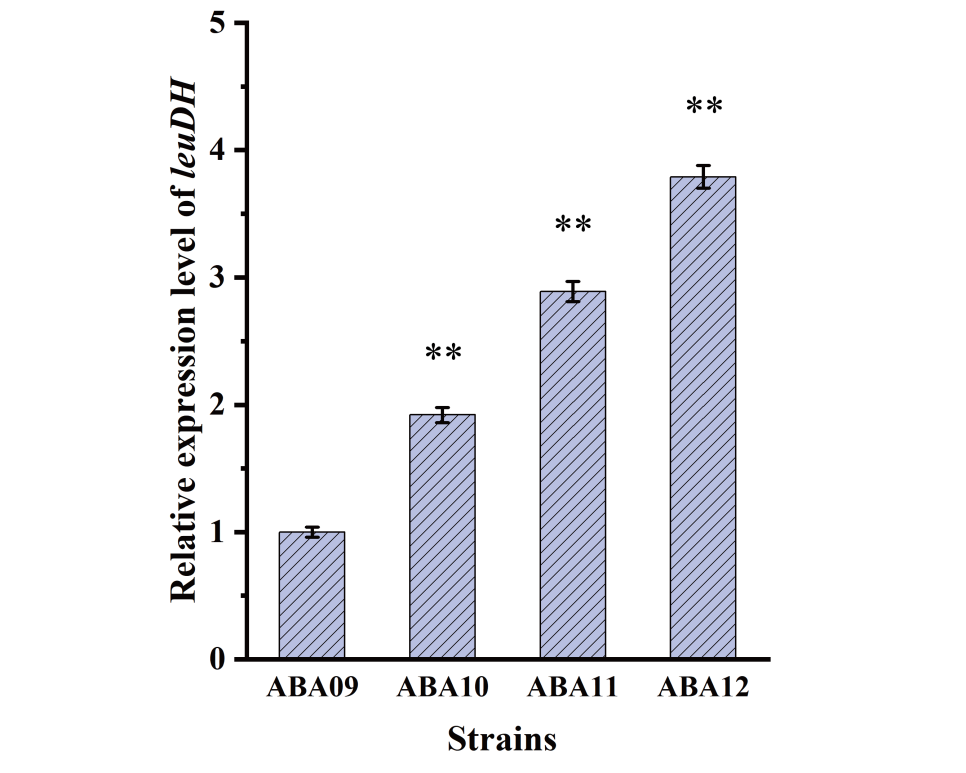
**
